# Vibrational circular dichroism spectroscopy for probing the expression of chirality in mechanically planar chiral rotaxanes†

**DOI:** 10.1039/d0sc02485f

**Published:** 2020-07-23

**Authors:** Mark A. J. Koenis, C. S. Chibueze, M. A. Jinks, Valentin P. Nicu, Lucas Visscher, S. M. Goldup, Wybren J. Buma

**Affiliations:** Van 't Hoff Institute for Molecular Sciences, University of Amsterdam Science Park 904 1098 XH Amsterdam The Netherlands W.J.Buma@uva.nl; Department of Chemistry, University of Southampton University Road, Highfield Southampton SO17 1BJ UK s.goldup@soton.ac.uk; Department of Environmental Science, Physics, Physical Education and Sport, Lucian Blaga University of Sibiu loan Ratiu Street, Nr. 7-9 550012 Sibiu Romania; Amsterdam Center for Multiscale Modeling, Section Theoretical Chemistry, Faculty of Sciences, Vrije Universiteit Amsterdam De Boelelaan 1083 1081 HV Amsterdam The Netherlands; Institute for Molecules and Materials, FELIX Laboratory, Radboud University Toernooiveld 7c 6525 ED Nijmegen The Netherlands

## Abstract

Mechanically interlocked molecules can exhibit molecular chirality that arises due to the mechanical bond rather than covalent stereogenic units. Developing applications of such systems is made challenging by the absence of techniques for assigning the absolute configuration of products and methods to probe how the mechanical stereogenic unit influences the spatial arrangements of the functional groups in solution. Here we demonstrate for the first time that Vibrational Circular Dichroism (VCD) can be used to not only discriminate between mechanical stereoisomers but also provide detailed information on their (co)conformations. The latter is particularly important as these molecules are now under investigation in catalysis and sensing, both of which rely on the solution phase shape of the interlocked structure. Detailed analysis of the VCD spectra shows that, although many of the signals arise from coupled oscillators isolated in the covalent sub-components, intercomponent coupling between the macrocycle and axle gives rise to several VCD bands.

## Abstract 2

Through the looking glass: VCD spectroscopy provides unique insight into how a chiral mechanical bond imposes shape on rotaxanes in solution and allows their absolute configuration to be determined.

## Graphical abstract

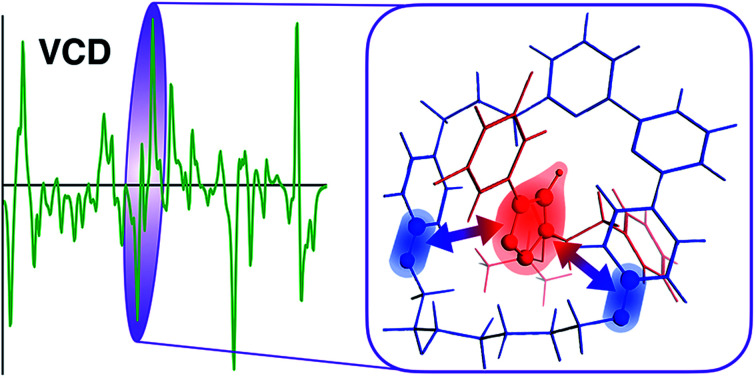

## Introduction

Mechanically interlocked molecules (MIMs)^1^ such as catenanes and rotaxanes have received tremendous amounts of interest over the past few decades for a range of applications.^2^ One of the primary reasons for this is that the mechanically bonded architecture allows for novel approaches to molecular switches and machines,^3^ an insight that culminated in the award of the 2016 Chemistry Nobel Prize to Stoddart and Sauvage, alongside Feringa.^4^ However, another intriguing property of MIMs that was recognized over 50 years ago,^5^ their ability to display chirality even in the absence of covalent stereochemistry,^6^ has been investigated much less extensively.

The first examples of enantiopure “mechanically chiral”^7^ molecules, a topologically chiral catenane and a mechanically planar chiral rotaxane, were reported by Sauvage and Vögtle respectively, in collaboration with Okamoto.^8,9^ Since these pioneering reports however, progress has been slow, with limited examples of the influence of mechanical stereogenic units demonstrated, including controlling the conformations of polymers,^10^ sensing of small chiral molecules,^11^ expression of stereodynamic behaviour,^12^ and catalysis.^13,14^ Key barriers to progress in the applications of mechanically chiral molecules are the difficulty of obtaining enantiopure samples^42^ and the subsequent stereochemical characterization of the products. To date, most reports make use of chiral stationary phase high-performance liquid chromatography (CSP-HPLC) for the separation of enantiomers, necessarily limiting the scale on which they can be produced, and in many cases, the absolute stereochemistry of the products remains unassigned.

To address the synthetic problem, Goldup and co-workers have developed scalable auxiliary approaches to mechanically planar chiral rotaxanes^15^ and topologically chiral catenanes.^16^ In contrast, methods to assign the absolute stereochemistry of the products remain limited and largely rely on single crystal X-ray diffraction (SCXRD),^15–17^ which requires suitable crystals and, typically, an internal stereochemical reference. Chiral spectroscopic methods would provide an ideal solution to the assignment problem and indeed, a great many mechanically chiral molecules have been analysed by electronic circular dichroism (ECD).^8,9^ However, the difficulties associated with accurately calculating the electronically excited states of large, flexible, MIMs^18^ means that caution should be exercised in assigning their absolute configuration on the basis of ECD measurements alone.

Vibrational Circular Dichroism (VCD), the relatively less well-known equivalent of ECD in the infrared spectral region,^19^ has the key advantages of much higher spectral resolution and relatively straightforward modelling of spectra as only the electronic ground state needs to be considered. VCD is therefore well suited to the study of complex molecular systems and, importantly, can provide detailed structural information in solution, because VCD signals depend strongly on the relative spatial arrangement of chromophores within the molecule; intense signals often arise from coupling between vibrational modes in two different fragments *via* the General Coupled Oscillator (GCO) mechanism,^20^ the vibrational counterpart to exciton–exciton interactions in ECD.^19*a*,21^ Thus, in combination with computational modelling, VCD can provide detailed information about the solution-state conformation of molecules, something that ECD, with its lower resolution and more challenging computational analysis, and SCXRD, which provides only information in the solid state, cannot achieve.

Here we report the first application of VCD to the study of mechanically planar chiral rotaxanes, employing methods that we recently developed for the analysis of highly flexible systems.^22^ We show that the computed VCD spectra are in good agreement with those obtained experimentally and thus that the configuration determined by VCD agrees with that from X-ray diffraction.^15*b*^ Using a combined experimental and computational approach, we determined the origin of the measured signals and how they arise due to mechanical stereogenic element, and so gain insight into the (co)conformation of these unusual chiral systems. Our results suggest that VCD analysis is suitable for accurately determining the absolute mechanical configuration in unknown systems and provides detailed structural information of particularly use in the development of mechanically chiral molecules for enantioselective applications. One aspect that comes prominently forward from this is that the chiroptical properties of such MIMs are more than the sum of their individual parts.

## Results and discussion

**Fig. 1:**
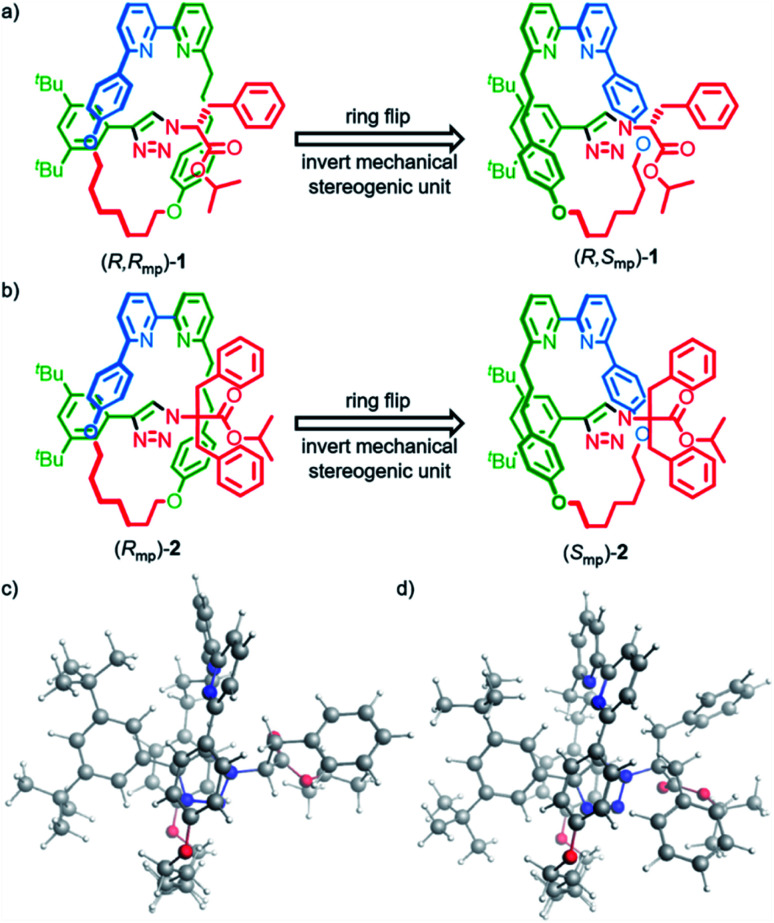
Rotaxanes (a) (*R*,*R*_mp_)-**1** and (b) (*R*_mp_)-**2** whose mechanical stereochemistry arises due to the orientation of the macrocycle relative to the axle and the corresponding isomers obtained by inverting the mechanical stereogenic unit. Lowest-energy conformations calculated for (c) (*R*,*R*_mp_)-**1** and (d) (*R*_mp_)-**2**.

Rotaxanes (*R*,*R*_mp_)-**1**, (*S*,*S*_mp_)-**1**, (*R*_mp_)-**2** and (*S*_mp_)-**2** (Fig. 1) were synthesized as previously reported^15*b*^ and their vibrational absorption (VA) and VCD spectra were recorded and compared with those obtained by computational modelling. To properly model and interpret VCD spectra, accurate conformational searches are needed to ensure that all accessible conformations are considered. For relatively big and often flexible systems like rotaxanes this is especially challenging due to sheer number of available conformations and the computational cost of optimizing their geometries.^23^

In brief (for full details see the ESI†), a conformational search was performed for rotaxanes (*R*,*R*_mp_)-**1** and (*R*_mp_)-**2** using the Macromodel program in the Schrödinger software suite (v11.9)^24,25^ by generating 10 000 starting structures using 100 steps per rotational bond and optimizing these structures with the OPLS3E forcefield. Conformations within 10 kcal mol^−1^ of the lowest energy structure were subsequently optimized with the Amsterdam Density Functional (ADF2019) software package using the density functional based tight-binding (DFTB3) method, an approximate quantum chemical method of lower computational cost than DFT, together with the 3ob-3-1 parameter set.^26–29^ This yielded 687 and 709 conformations for (*R*,*R*_mp_)-**1** and (*R*_mp_)-**2**, respectively.

Conformations within 2 kcal mol^−1^ of the lowest-energy DFTB3 conformation were subjected to DFT analysis (BP86/TZP) using the ExactDensity option in ADF.^30–32^ The remaining structures were clustered into 30 groups using a distance matrix based on the interatom distance differences between structures and the agglomerative hierarchical clustering function with average linkage in MATLAB (see ESI†).^33^ The two conformers with lowest DFTB3 energy of each group were optimized with DFT and, if their energy was found to be within 2 kcal mol^−1^ of the lowest-energy conformer, all the other structures of that cluster were optimized by DFT. In total, 220 and 261 conformations were optimized with DFT for rotaxanes (*R*,*R*_mp_)-**1** and (*R*_mp_)-**2**, respectively.

The VA and VCD spectra of all structures within 2 kcal mol^−1^ of the lowest-energy conformation were computed using DFT,^34^ and modelled spectra produced using Boltzmann weighting. The computed VA and VCD intensities were convoluted with a Lorentzian function using a full-width at half-maximum of 8 cm^−1^ while the computed frequencies were scaled using the method developed by Shen *et al.*^35^

**Fig. 2:**
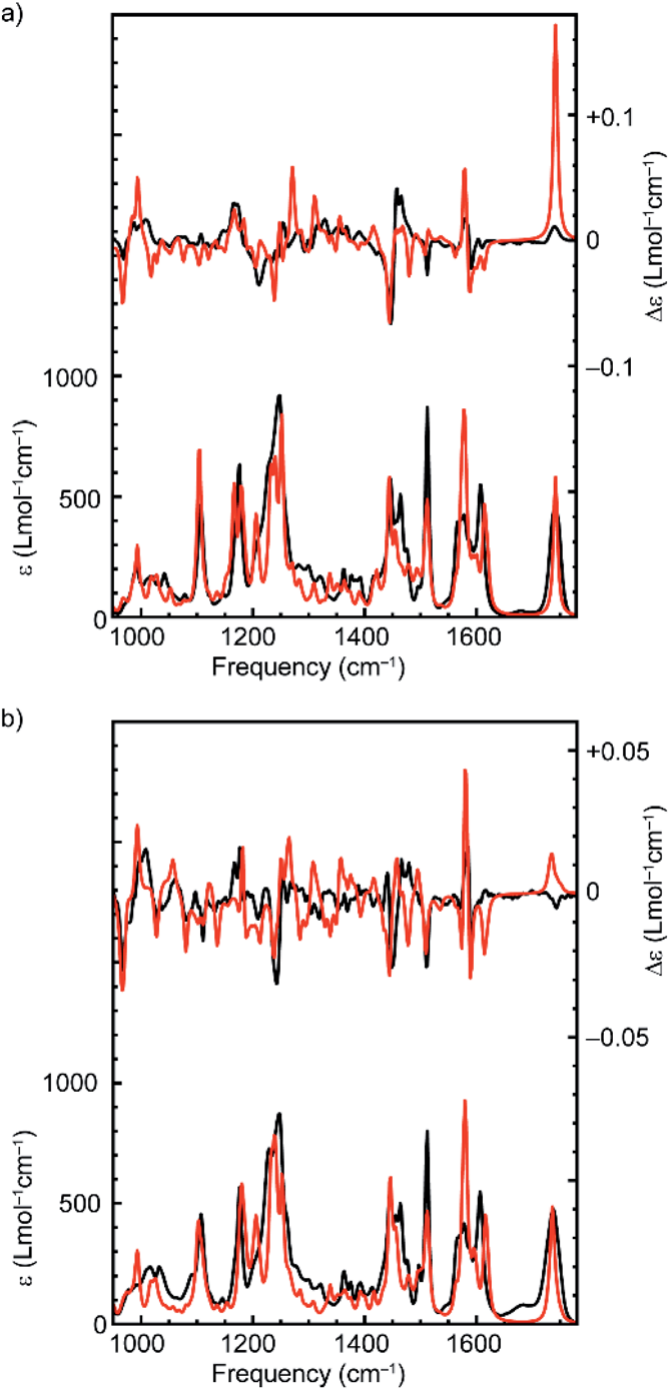
Comparison between the experimental (black) and Boltzmann weighted computed (red) VA (bottom) and VCD (top) spectra of (a) (*R*,*R*_mp_)-**1** and (b) (*R*_mp_)-**2**. Experimental spectra (CDCl_3_, 0.15 M and 0.09 M for (a) (*R*,*R*_mp_)-**1** and (b) (*R*_mp_)-**2** respectively) were obtained over 48 h in a 75 μm BaF_2_ sample cell. The spectrum of pure CDCl_3_ was used as a baseline. The enantiomeric VCD spectra have been subtracted from each other to remove baseline artefacts.

The computed VA spectra of (*R*,*R*_mp_)-**1** and (*R*_mp_)-**2** (Fig. 2) are in good agreement with the experimental spectra, which is confirmed quantitatively by SimIR values^35^ for the region from 950 to 1750 cm^−1^ of 0.82 and 0.80 for (*R*,*R*_mp_)-**1** and (*R*_mp_)-**2**, respectively. The similarity between the experimental and computed VCD spectra of both rotaxanes is lower, with SimVCD values^35^ of 0.22 and 0.29 for (*R*,*R*_mp_)-**1** and (*R*_mp_)-**2**, respectively. This is in part due to the complexity and flexibility of the system, but also specifically because of the large difference observed between experimental and simulated spectra in the carbonyl stretching region at 1750 cm^−1^. If this band is not taken into account significantly better agreement is obtained, in particular for (*R*,*R*_mp_)-**1** (SimVCD = 0.33 and 0.30 for (*R*,*R*_mp_)-**1** and (*R*_mp_)-**2**, respectively).

Rotaxanes (*R*,*R*_mp_)-**1** and (*R*_mp_)-**2** have many low-energy conformers. Due to the intrinsic uncertainty in their relative energies it is important to test whether it is possible to generate a better fit by re-weighting the Boltzmann average used to generate the calculated spectra.^22^ Using a genetic fitting algorithm that optimizes the energies of the conformations utilizing an energy uncertainty of 1 kcal mol^−1^ led to improved fits to the experimental VCD data of +0.40 and +0.41 for (*R*,*R*_mp_)-**1** and (*R*_mp_)-**2** respectively. More importantly, using the same approach, we were unable to generate satisfactory fits to the experimental data of enantiomeric structures (*S*,*S*_mp_)-**1** and (*S*_mp_)-**2**; applying the genetic fitting algorithm yielded overlaps of just −0.28 and −0.19 respectively (see Fig. S1† for spectra simulated with optimized Boltzmann weights). The difference in SimVCD values for the enantiomers is significant and demonstrates that it is highly unlikely that the calculated data, within the uncertainty of the computed energies, can lead to an incorrect stereochemical assignment, strongly supporting the proposal that VCD is a powerful means to determine the absolute configuration of these MIMs.

The experimental VA spectra of rotaxanes (*R*,*R*_mp_)-**1** and (*R*_mp_)-**2** are nearly identical (SimIR = 0.98), in keeping with the only difference between the two structures being an additional benzyl group in the latter. When comparing the experimental VCD spectra of the two rotaxanes we see that the most intense bands have similar signs (see Fig. S2† for a direct comparison); both compounds have positive bisignate bands (−/+) at 1000 and 1450 cm^−1^, negative bisignate bands at 1150 and 1575 cm^−1^ and a single negative band at 1500 cm^−1^. Importantly, however, we find that the signal intensities in the VCD spectrum of (*R*_mp_)-**2** are notably smaller than for (*R*,*R*_mp_)-**1**, especially for the bisignate bands at 1150 and 1450 cm^−1^. Further analysis of the conformational heterogeneity of rotaxanes (*R*,*R*_mp_)-**1** and (*R*_mp_)-**2** suggests that this difference is caused, at least in part, by the increased conformational degrees of freedom introduced by the additional benzyl group in (*R*_mp_)-**2**, which adds two rotatable bonds to the system; (*R*,*R*_mp_)-**1** is predicted to have 28 unique conformations within 2 kcal mol^−1^ of the lowest-energy conformation whereas (*R*_mp_)-**2** is predicted to have 71 conformations in the same energy interval. This increase in conformational heterogeneity leads to more spectra being included in the Boltzmann-weighted average, which in turn results in an increase in spectral averaging and thus reduced signal intensity (Fig. S3†). Conversely, when only the lowest-energy structures of (*R*,*R*_mp_)-**1** and (*R*_mp_)-**2** are considered, comparable VCD signal intensities are calculated (Fig. S3c†). This suggests that the covalent stereocentre of (*R*,*R*_mp_)-**1** has a relatively limited influence on the VCD spectrum. This suggestion finds further support in the calculated VCD spectrum of (*S*,*R*_mp_)-**1** (Fig. S4†) which shows that inversion of the covalent stereocentre does not lead to significant inversions in the VCD spectrum. In this regard the carbonyl stretching band is particularly noteworthy as it does not change sign, even though this mode is localised on a group directly attached to the stereogenic carbon atom whose configuration is inverted.

**Fig. 3:**
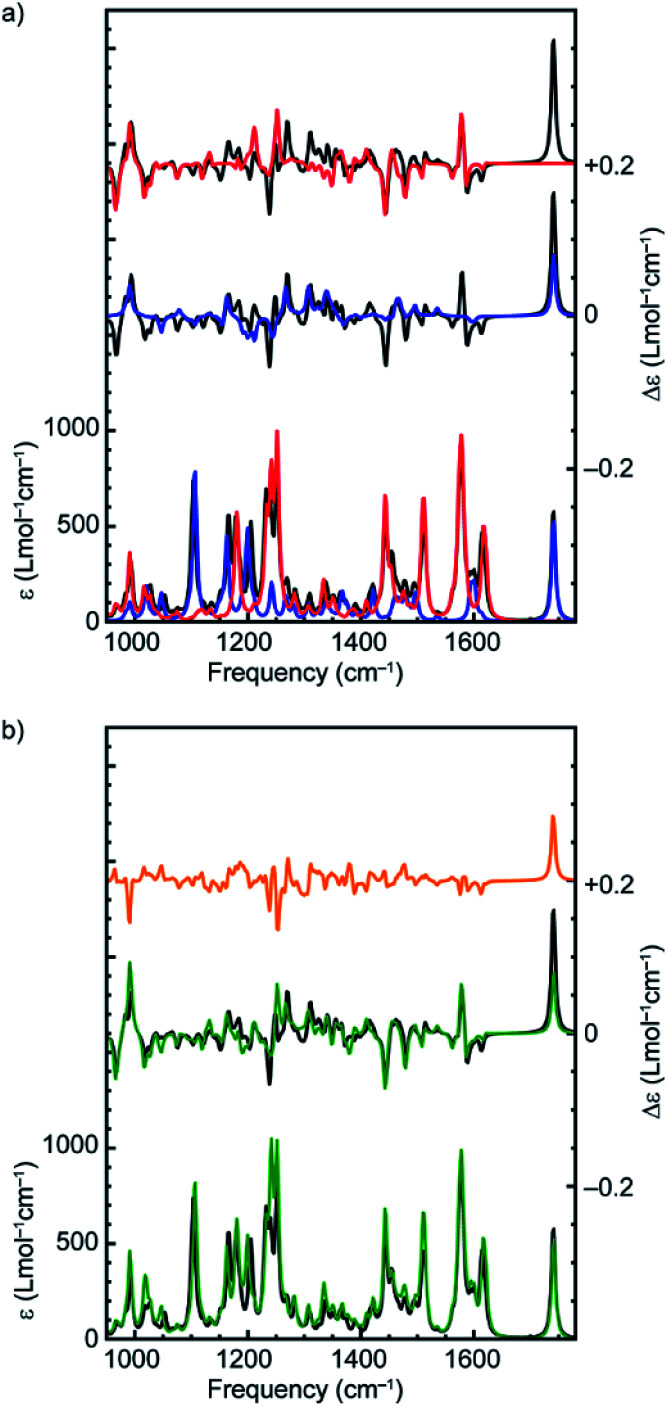
(a) Comparison between the calculated VA and VCD spectra of the lowest-energy conformer of (*R*,*R*_mp_)-**1** (black), and the isolated macrocycle (red) and axle (blue) in the same conformation as found in the rotaxane. (b) Comparison between the calculated VA and VCD spectra of the lowest-energy conformer of (*R*,*R*_mp_)-**1** (black), the sum of the spectra of the isolated macrocycle and axle (green), and the difference between these sum spectra and the spectrum of the complete rotaxane (orange).

The suggestion that the covalent stereogenic unit of rotaxane **1** plays, at most, a minor role in its chiroptical properties, raises the interesting question of which molecular features ultimately determine the signs and intensities of the bands in the VCD spectra of these MIMs. In particular, we were interested to determine to what extent the axle and macrocycle can be considered as independent moieties embedded within the chiral environment created by the mechanical bond. To this purpose, we extracted the covalent sub-components from the lowest-energy conformation of (*R*,*R*_mp_)-**1** and (*R*_mp_)-**2** (Fig. 1), calculated their VCD spectra without further geometry optimization and compared these calculated spectra with those of the complete rotaxane (Fig. 3a and Fig. S5† respectively).,^36^ Surprisingly, in both cases, the more intense VCD bands appear to originate from the macrocycle alone, even in the case of (*R*,*R*_mp_)-**1** whose axle contains a covalent stereocentre but only contributes significantly to the 1100–1300 cm^−1^ region (where the ester and triazole groups are active) and the carbonyl stretch band at 1750 cm^−1^.

However, the VCD spectrum of (*R*,*R*_mp_)-**1** cannot be considered to simply be the sum of the spectra arising from the individual sub-components. Strikingly, the calculated intensity of the carbonyl stretch band is only half as large for the isolated axle as compared to that obtained for (*R*,*R*_mp_)-**1** (+134 instead of +278 × 10^−44^ esu^2^ cm^2^). A GCO analysis^20^ (Table 1) of this mode indicates that the entire VCD signal originates from the ester group, indicating that there is no coupling with the macrocycle for this particular vibration. The VCD intensity differences observed between axle and rotaxane are therefore attributed to intramolecular electrostatic interactions between macrocycle and carbonyl group. Such intermolecular interactions are generally more difficult to properly describe from a computational point of view and might thus explain the differences between theory and experiment observed for this mode (Fig. 2).

**Table 1:** Rotational strengths and decomposition for the intense (*R*_01_ ≥ 100) VCD modes of the lowest-energy conformation of (*R*,*R*_mp_)-**1** with macrocycle and axle selected as fragments^a^

Frequency	*R*_01_	*R* ^IF,MC^_01_	*R* ^IF,axle^_01_	*R* ^GCO^_01_
948	−115	−156	+0	+1
974	+157	+130	+2	+26
1180	−121	+0	−90	−30
1222	−225	−185	−1	−41
1232	+149	+29	−63	+183
1235	−106	+145	−17	−234
1252	+139	+1	+114	+24
1424	−116	−94	−0	−21
1713	+278	−0	+274	+4

a *R* ^IF,MC^_01_ and *R*^IF,axle^_01_ are the rotational strengths arising from the macrocycle and axle individually. *R*^GCO^_01_ is the contribution arising from the vibrational coupling interaction between macrocycle and axle. Frequencies are given in cm^−1^, rotational strengths in 10^−44^ esu^2^ cm^2^.

In addition, a number of VCD signals arise from inter-component vibrational couplings as becomes clear when the individual spectra for the macrocycle and axle are summed and compared with the calculated VCD spectrum of the complete rotaxane; several intense bands in the 1200–1300 cm^−1^ region appear in the VCD spectrum of the rotaxane which are not present in sum of the individual component spectra (Fig. 3b). To obtain a quantitative picture of how important this intercomponent vibrational coupling is and to understand its origin we performed a GCO analysis using the GUI implementation of VCD tools in ADF,^20,37^ with the axle and macrocycle as coupling fragments. The computed interaction terms, *R*^GCO^_01_, for the intense bands in the spectrum show a similar picture to that concluded graphically from the spectra, in that most of the intense VCD bands originate from the macrocycle component (Table 1, Fig. S6†). This is perhaps unsurprising as the macrocycle contains several pairs of near-identical functional groups (*e.g.* pyridine, and phenoxy ether groups); because these groups have similar vibrational modes, they are prone to being strongly coupled. However, several bands clearly cannot be localized on either macrocycle or axle but find their origin in the coupling between vibrational modes in both components. Interestingly, the strongest interaction between the macrocycle and the axle is not associated with the phenyl groups of the macrocycle which engage in π–π interactions with the triazole ring. Instead, coupling between the C–N stretch mode of the triazole group of the axle and the C–O stretch mode of the two ether groups of the macrocycle results in a strong bisignate band around 1234 cm^−1^ (Table 1).

**Fig. 4:**
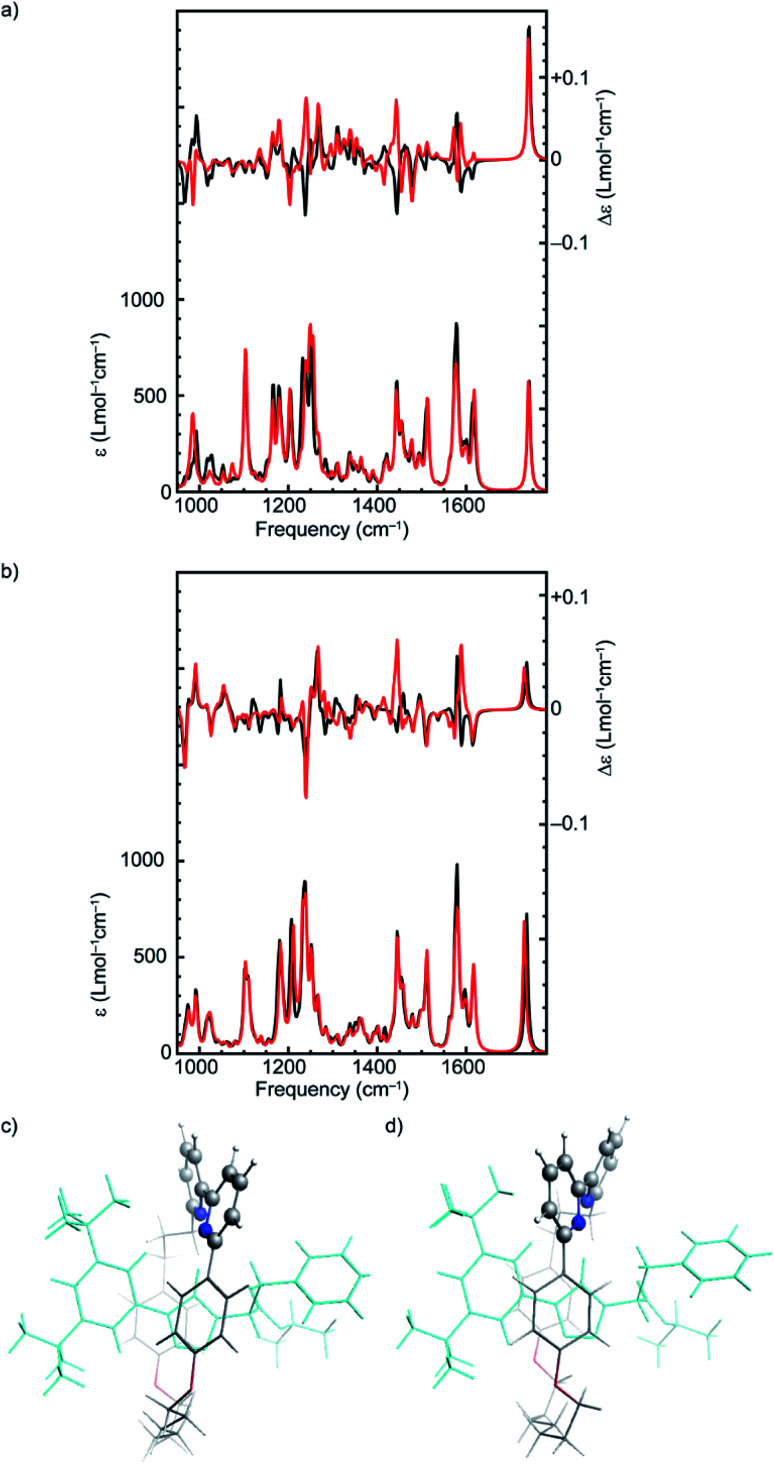
Effects of changing the axial chirality of the two pyridine groups of the macrocycle on the VA and VCD spectra of (*R*,*R*_mp_)-**1** and (*R*_mp_)-**2**. Panels (a) and (b) compare VA and VCD spectra of the lowest-energy conformer (black) and the lowest-energy conformer with the bipyridine axial chirality inverted (red) for (*R*,*R*_mp_)-**1** and (*R*_mp_)-**2**, respectively. Panels (c) and (d) display the structures of the lowest-energy conformer of (*R*,*R*_mp_)-**1** and the same conformer with inverted bipyridine axial chirality.

In order to come to a better understanding of the relationship between the stereogenic units and the conformations that result from them and give rise to the VCD signals, we identified a higher-energy conformer of (*R*_mp_)-**2** whose bisignate band at 1575 cm^−1^ is inverted with respect to spectra of the other 70 conformers (Fig. S2†). By inspection of the inverted modes using a GCO analysis it became clear that these VCD signals arise from the coupling of the two pyridine groups, which is critically dependent on their orientation with respect to each other. For the specific conformer with an inverted bisignate band the dihedral angle between the two groups (N–C–C–N) is +26° while for all other low-energy conformers it lies between −13° and −30° (Fig. 4c and d). This modification inverts the absolute configuration of the flexible bipyridine rotamer and explains the sign inversions observed in the calculated VCD spectrum. Similar inverted dihedral angles and an associated inverted VCD signal were found for other energetically higher-lying conformations of rotaxane (*R*_mp_)-**2**, as well as for higher-lying conformations of rotaxane (*R*,*R*_mp_)-**1**. We conclude that for these rotaxanes the mechanically planar chiral stereogenic unit leads to a preference for one configuration of the flexible, conformationally chiral bipyridine unit.

The observation that several intense bands in the VCD spectra ultimately originate from this rather weak preference in the conformation of the macrocycle has major consequences for the reliability of the enantiomeric assignment of these rotaxanes. To demonstrate this more clearly, we attempted to isolate the effect of the axial conformational chirality of the bipyridine group by replacing the macrocycle of the lowest-energy conformer of (*R*,*R*_mp_)-**1** and (*R*_mp_)-**2** with the macrocycle of the lowest-energy conformer with an inverted dihedral angle between the pyridine rings. The resulting spectra (Fig. 4) confirm the expected outcome; for both (*R*,*R*_mp_)-**1** and (*R*_mp_)-**2** several intense VCD bands have changed sign. These include the strong band at 1424 cm^−1^ which derives its VCD intensity from the bipyridine unit of the macrocycle (Table 1) but also the bisignate feature at 1575 cm^−1^. Similarly, we find the negative strong bands at 948 and 1222 cm^−1^ and the positive band at 974 cm^−1^ critically depend on the orientation of the two ether groups as these bands stem from the coupling between them.

Finally, in keeping with our previous studies,^38^ we find that ECD is complementary to VCD, providing a powerful means to avoid an incorrect assignment. The main features in the ECD spectrum of (*R*,*R*_mp_)-**1** and (*R*_mp_)-**2** appear at 300 nm,^15*b*^ consistent with the exciton coupling of the two pyridine rings. Using the empirical quadrant approach, it is simple to demonstrate that the dihedral N–C–C–N angle found by modelling is consistent with the observed ECD. Thus, both calculated VCD and ECD are in agreement with the experimental data. It should be noted, however, that it is not possible to assign the absolute mechanical stereochemistry of (*R*,*R*_mp_)-**1** and (*R*_mp_)-**2** simply by determining the configuration of this conformational stereochemical unit by ECD as there is no obvious direct relationship between the mechanical configuration and the conformation of the bipyridine unit. Detailed modelling is required to determine the preferred conformation of the macrocycle and, once this has been carried out, the agreement of the experimental and calculated VCD spectra then gives access to detailed information on key aspects of the co(conformations) of these chiral MIMs, which is simply not possible using ECD alone. We emphasize that for application purposes the combined use of ECD and VCD has distinct advantages as VCD can provide assignment of absolute mechanical stereochemistry and detailed information about molecular (co)conformation, while ECD can be used conveniently for rapid quality control purposes.

## Conclusions

We have presented a first in-depth VCD study of mechanically planar chiral rotaxanes as prototypical examples of mechanically chiral molecules and demonstrated that a combined computational/experimental approach can provide detailed insight into the molecular aspects that determine how their stereochemistry manifests spectroscopically. Detailed analysis of the source of intensity of bands in their VCD spectra reveals that many of the signals arise from coupled modes within the achiral macrocycle through conformational biasing of this component by the mechanical stereogenic unit, and that the covalent stereocentre of (*R*,*R*_mp_)-**1** has far less influence. In addition to these intra-component bands, strong VCD signals also arise from coupled modes between the subcomponents, demonstrating that, from a VCD point of view, these rotaxanes are more than the sum of their parts. To date, the stereochemistry of such assemblies in solution has been studied qualitatively using methods like ECD and, where it has been achieved, absolute stereochemistry has typically been assigned crystallographically. Here we have shown that VCD has the potential to both determine the absolute mechanical configuration of these challenging structures and probe their preferred (co)conformation in solution, where the majority of their current applications are found.

Although these results are promising, some caveats must be highlighted. First, careful attention must be paid to the conformational search used to generate the modelled VCD spectra as the comparison between experiment and theory is an essential feature of the analysis. This is not trivial since these molecules are large and their conformations depend on relatively subtle intercomponent interactions.^37,39^ However, with the advent of efficient, modern computational methods and powerful, affordable computers, such challenging structures are now reasonable targets for computational analysis. Second, our results to date are confined to a single class of mechanically planar chiral rotaxane, those derived from an active template^40^ approach using a chiral auxiliary strategy,^15^ as they are one of the few examples whose absolute configuration has been unambiguously characterised. However, although insights regarding the conformational stereogenic units of (*R*,*R*_mp_)-**1** and (*R*_mp_)-**2** are specific to these structures, the general point that VCD can provide information about molecular shape by probing intra- and inter-component coupled modes remains and is well demonstrated in other areas.^19*b*^

Thus, we are confident that by demonstrating the power of VCD in these specific examples, we have paved the way for its application to mechanically chiral rotaxanes and catenanes more generally. Future work will focus on expanding our methods to a range of structural motifs in order to identify the key stumbling blocks to using VCD as a routine tool for the assignment of mechanical configuration in rotaxanes and catenanes, and the detailed analysis of the expression of their mechanical stereochemistry. We believe the insights that can be obtained by VCD will assist in the design of chiral MIMs for a range of applications, including sensing,^11^ catalysis^13^ and materials chemistry.^41^

## Conflicts of interest

There are no conflicts to declare.

## Supplementary Material

SC-011-D0SC02485F-s001
