# Supplementary material for: Vibrational circular dichroism spectroscopy for probing the expression of chirality in mechanically planar chiral rotaxanes
Source: Chem Sci. 2020 Jul 23;11(32):8469–75. doi: 10.1039/d0sc02485f (PMC8163398; doi:10.1039/d0sc02485f)
Supplement: SC-011-D0SC02485F-s001 [file SC-011-D0SC02485F-s001.pdf]

Electronic Supplementary Material (ESI) for Chemical Science.
This journal is © The Royal Society of Chemistry 2020

Supporting Information

Vibrational Circular Dichroism Spectroscopy for Probing the Expression of Chirality in Mechanically Planar Chiral Rotaxanes

Mark A. J. Koenis,[a] C. Chibueze,[a] M. A. Jinks,[b] Valentin P. Nicu,[c] Lucas Visscher,[d] S.M. Goldup,[*,b] and Wybren J. Buma[*,a,e]

[a]Van 't Hoff Institute for Molecular Sciences, University of Amsterdam, Science Park 904, 1098 XH Amsterdam, The Netherlands
[b]Department of Chemistry, University of Southampton, University Road, Highfield, Southampton, SO17 1BJ, UK
[c]Department of Environmental Science, Physics, Physical Education and Sport, Lucian Blaga University of Sibiu, loan Ratiu Street, Nr. 7-9, 550012 Sibiu, Romania
[d]Amsterdam Center for Multiscale Modeling, Section Theoretical Chemistry, Faculty of Sciences, Vrije Universiteit Amsterdam, De Boelelaan 1083, 1081 HV Amsterdam, The Netherlands
[e]Institute for Molecules and Materials, FELIX Laboratory, Radboud University, Toernooiveld 7c, 6525 ED Nijmegen, The Netherlands

E-mail: S.Goldup@soton.ac.uk; w.j.buma@uva.nl

General experimental

Rotaxanes (R,R_{mp})-**1**, (S,S_{mp})-**1**, (R_{mp})-**2** and (S_{mp})-**2** were synthesized using the method described by Goldup and co-workers.[1]

Experimental Spectra

Vibrational absorption (VA) and VCD spectra were recorded using a Biotools Chiral-2X spectrometer. All samples were measured for 48 h using a 75 μm BaF_2 sample cell. The rotaxanes were dissolved in $CDCl_3$ (0.15 M and 0.09 M for rotaxanes **1** and **2**, respectively). The spectrum of pure $CDCl_3$ was used as a baseline. The enantiomeric VCD spectra have been subtracted from each other to remove baseline artifacts.

Computational Modelling of Vibrational Circular Dichroism Spectra

Conformational search and DFTB optimisation: A conformational search was performed on rotaxanes (R,R_{mp})-**1** and (R_{mp})-**2** using the Macromodel program in the Schödinger software suite (v11.9).[2,3] This search was performed by generating 10,000 starting structures using 100 steps per rotational bond and a combination between bond rotations and low-frequency mode translations. The generated structures were optimized with the OPLS3E forcefield using the Conjugate Gradient method. The maximum atom deviation cut-off was set to 0.5 Å, the probability of a rotation/molecule translation to 0.5 Å and the minimum and maximum distance for low-mode moves to 3 and 6 Å. During the searches, each low energy conformation was identified multiple times (12 or more for (R,R_{mp})-**1**, 7 or more for (R_{mp})-**2**)). The generated conformers were filtered by hand to remove structures in which the macrocycle was disconnected from the axle by rotating one component through the other. Conformations within 10 kcal/mol of the lowest energy structure were subsequently optimized with the Amsterdam Density Functional (ADF2019) software package using the density functional based tight-binding (DFTB3) method together with the 3ob-3-1 parameter set.[4,5,6,7] This yielded 687 and 709 unique conformations for (R,R_{mp})-**1** and (R_{mp})-**2** respectively, making it computationally very costly to perform full density functional theory (DFT) calculations on all conformations.

Conformational clustering and DFT (BP86/TZP; ExactDensity option in ADF2019) optimisation of conformers: All conformations within 2 kcal/mol of the lowest-energy conformation based on the DFTB3 energies were optimised by DFT. To ensure low energy conformations were not missed, a distance matrix was computed for the remaining structures using the sum of the differences in pair-wise interatomic distances (excluding H atoms attached to carbon) between two structures. This distance matrix was then used to cluster the remaining conformations into 30 groups using the agglomerative hierarchical clustering function with average linkage in MATLAB.[8] The two conformers with lowest energy (DFTB3) of each group were optimized by DFT and, if the energy was found to be within 2 kcal/mol of the lowest-energy conformer found by DFT, all other structures in that cluster were also optimized using DFT. In total, 220 and 261 conformations were optimized using DFT for rotaxanes (R,R_{mp})-**1** and (R_{mp})-**2**, respectively.

VCD modelling: Finally, the VA and VCD spectra of all conformations within 2 kcal/mol of the lowest-energy conformation found by DFT were computed (DFT, TZP/BP86 level of theory using the ExactDensity option in ADF).[9,10,11,12] The generated spectra were then averaged using Boltzmann weighting. The computed VA and VCD intensities were convoluted with a Lorentzian function using a full-width at half-maximum of 8 cm^{-1} while the computed frequencies were scaled using the method developed by Shen *et al.*[13].

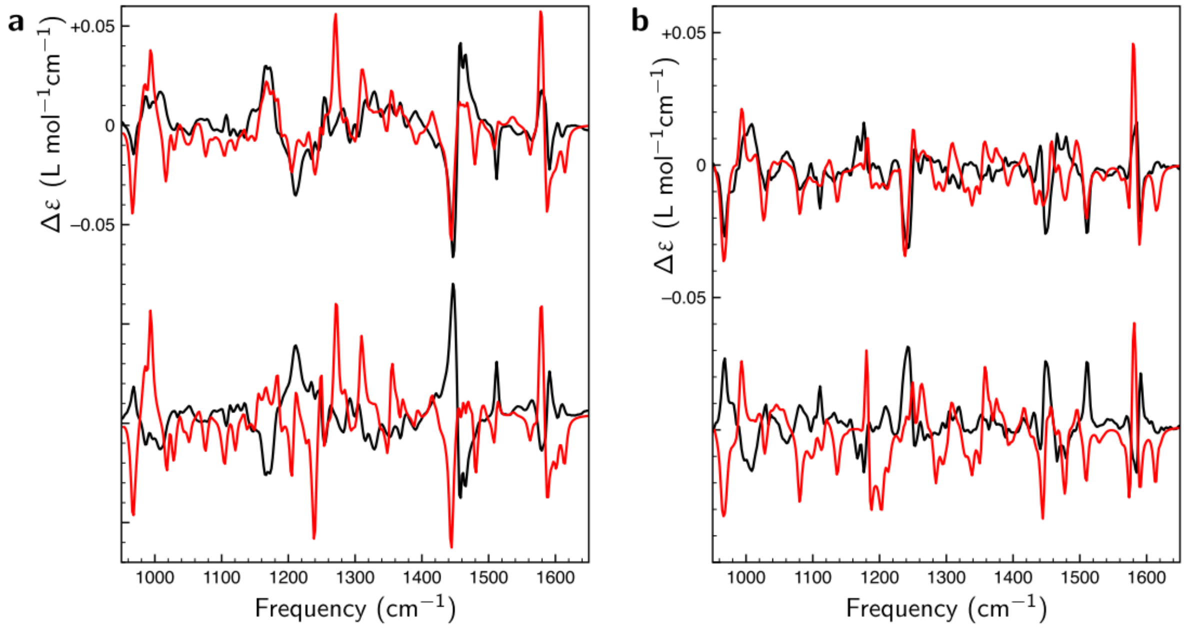

Figure S1: Comparison of experimental (black) and computed (red) VCD spectra using conformational Boltzmann weights optimized to the spectra of the correct enantiomer (top) and the opposite enantiomer (bottom), using a genetic fitting algorithm and a maximum energy deviation of 1 kcal/mol[14] for (a) (R,R_{mp})-**1** and (b) (R_{mp})-**2**.

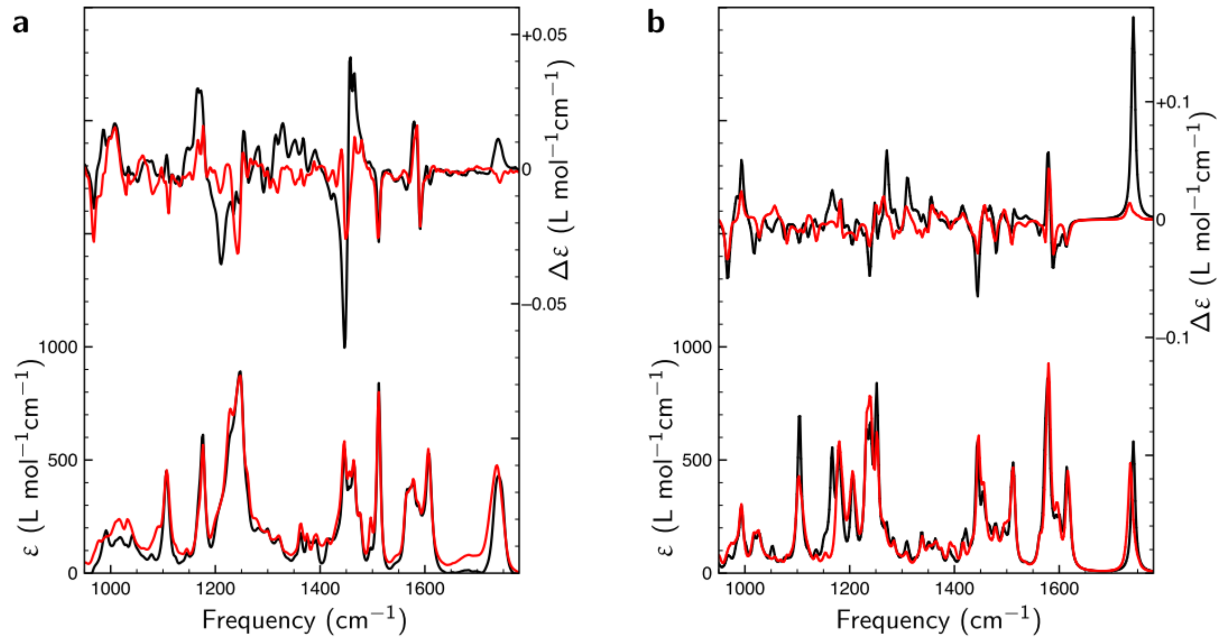

Figure S2: Comparison between VA (bottom) and VCD (top) spectra of (R,R_{mp})-**1** (black) and (R_{mp})-**2** (red). Panel (a) displays experimental spectra, panel (b) computed spectra.

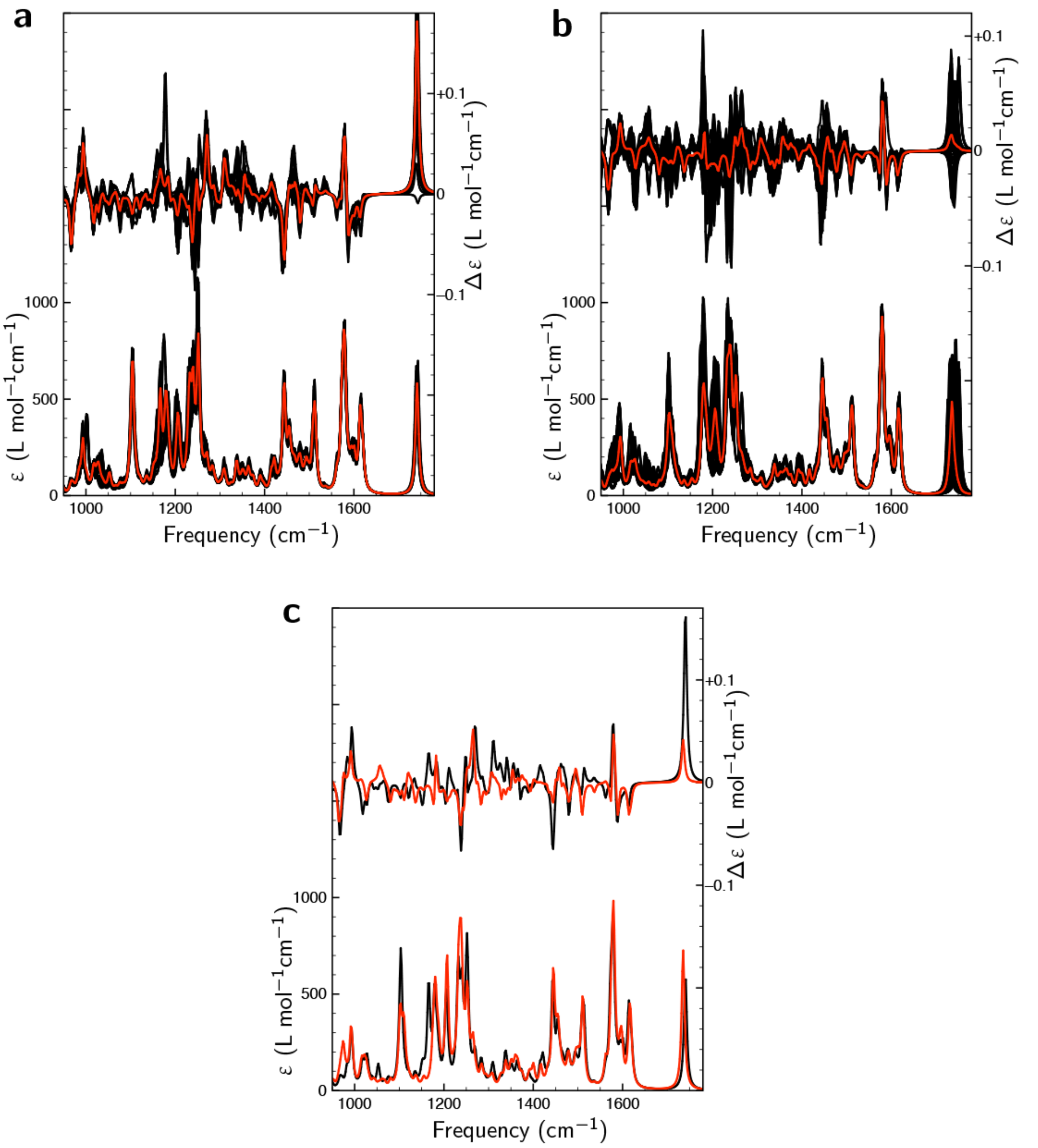

Figure S3: Comparison between VA and VCD spectra of low-energy conformations of rotaxanes (black) and their Boltzmann averaged spectra (red). Panel (a) displays computed spectra of (R,R_{mp})-**1** and panel (b) computed spectra of (R_{mp})-**2**. Panel (c) shows a comparison between the VA and VCD spectra of the lowest-energy conformers of (R,R_{mp})-**1** (black) and (R_{mp})-**2** (red).

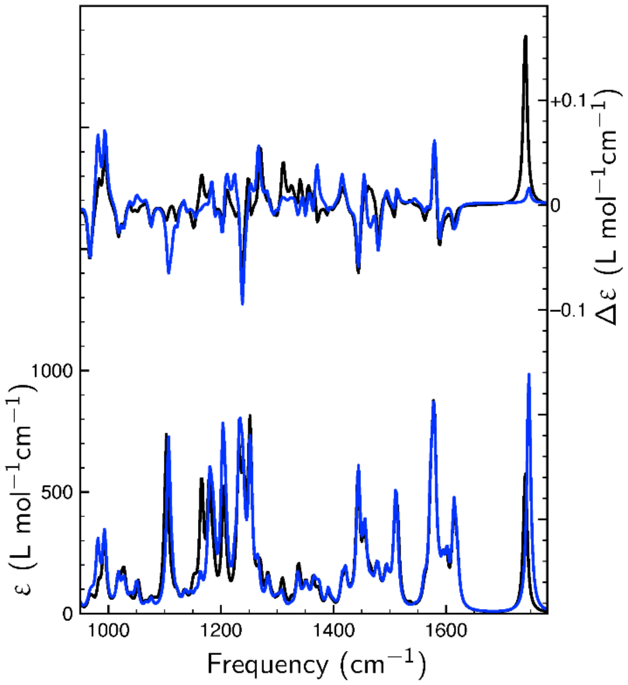

Figure S4: Comparison between the VA and VCD spectra of the lowest-energy conformer of (*R*,R_{mp})-**1** (black) and the spectra of the same conformer but with its chiral center inverted to form the (*S*,R_{mp})-**1** diastereomer (blue) (the H and Bn substituents were exchanged on the stereogenic C and the structure subsequently optimized to a minimum by DFT).

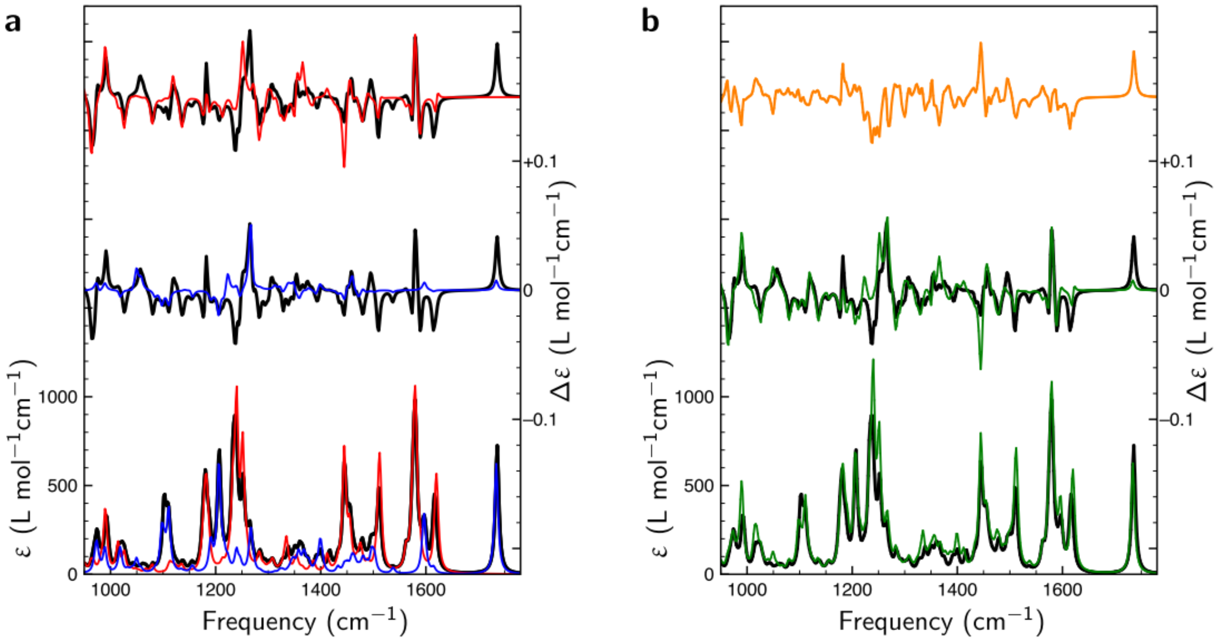

Figure S5: (a) Comparison between VA and VCD spectra of the lowest-energy conformer of (R_{mp})-**2** (black), as well as of its macrocycle (red) and thread (blue) separately. (b) Comparison between VA and VCD spectra of the lowest-energy conformer of (R_{mp})-**2** (black), the sum of the spectra of the macrocycle and the thread (green), and the difference between the sum spectra of the macrocycle and thread and the spectrum of the complete rotaxane (orange).

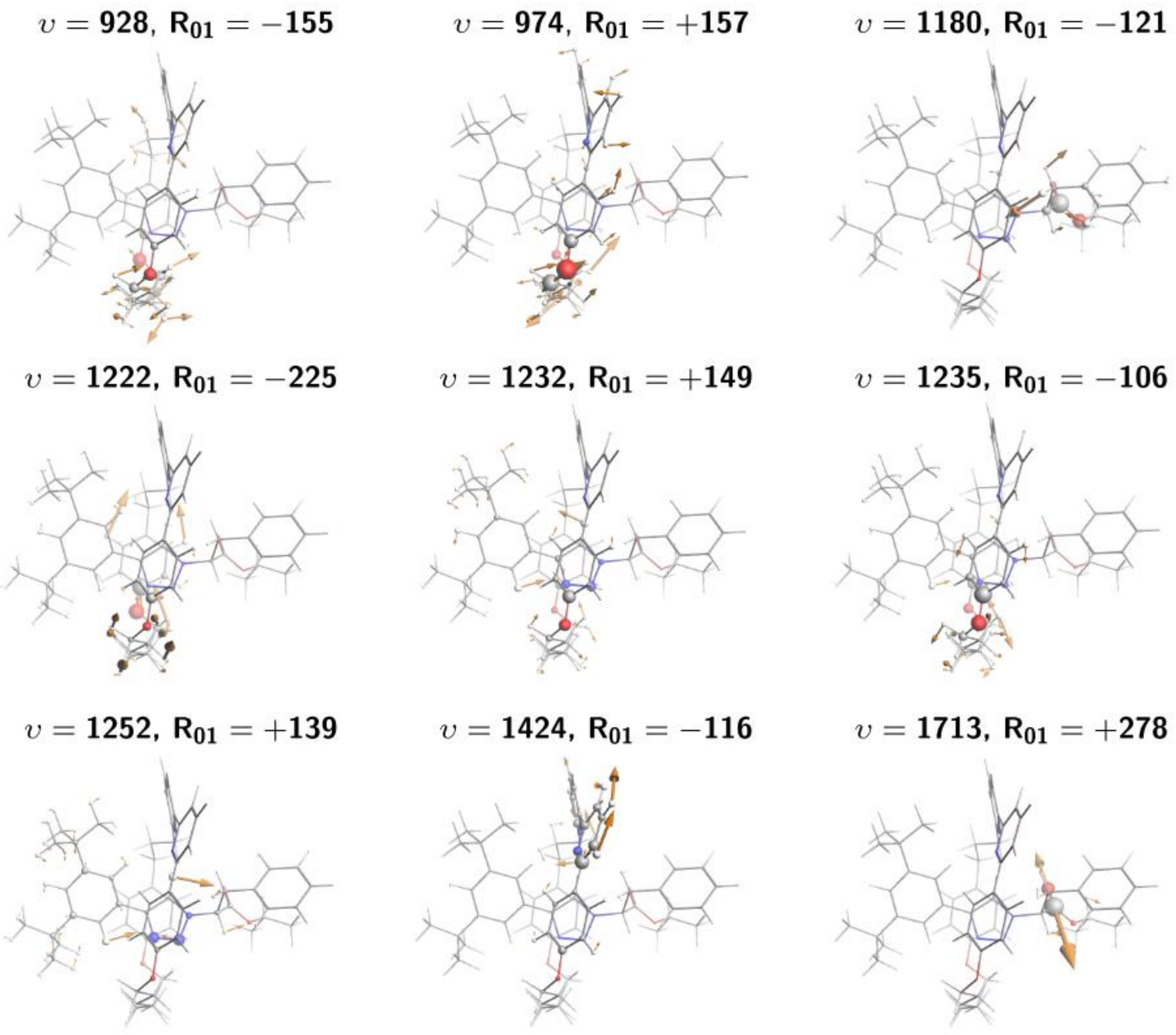

Figure S6: Nuclear displacement vectors (orange) of nine normal modes of the lowest energy conformer of (*R*,*R*$_{mp}$)-**1** with the highest computed rotational strength ($\mathbf{R_{01}}$) in the region between 900 and 1800 cm^{-1}. To illustrate the source of the VCD intensity of each mode, the radius of the atoms was scaled with the magnitude of the associated atomic EDTMs using the novel VCDtools implementation in ADF.[15] Frequencies *υ* are given in cm^{-1}, $\mathbf{R_{01}}$ in 10^{-44} esu^{2}cm^2.

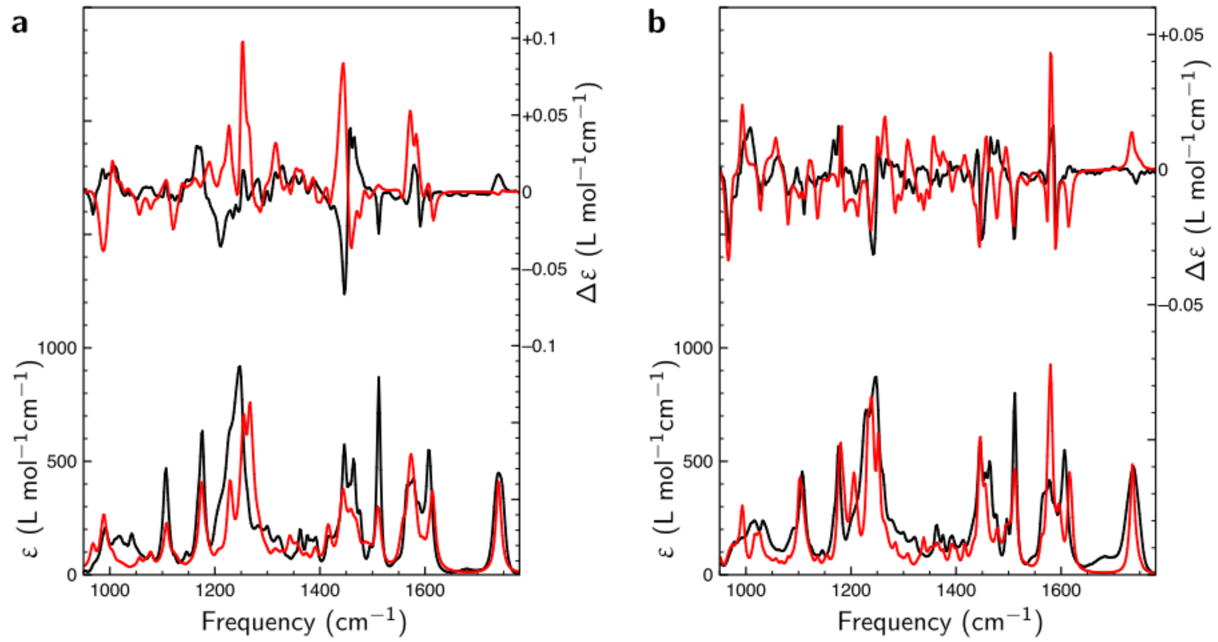

Figure S7: Comparison between experimental (black) and computed (red) VA (bottom) and VCD (top) spectra calculated using the DFT-D3-BJ dispersion term for (a) (R,R_{mp})-**1** and (b) (R_{mp})-**2**.

References

1 M. A. Jinks, A. de Juan, M. Denis, C. J. Fletcher, M. Galli, E. M. G. Jamieson, F. Modicom, Z. Zhang and S. M. Goldup, *Angew. Chem. Int. Ed.*, 2018, **57**, 14806-14810.
2 F. Mohamadi, N. G. J. Richards, W. C. Guida, R. Liskamp, M. Lipton, C. Caufield, G. Chang, T. Hendrickson and W. C. Still, *J. Comp. Chem.*, 1990, **11**, 440-467.
3 Schrödinger LLC, 2009. http://www.schrodinger.com.
4 Amsterdam Density Functional program. http://www.scm.com (accessed November 8, 2017).
5 G. te Velde, F. M. Bickelhaupt, E. J. Baerends, C. Fonseca Guerra, S. J. A. van Gisbergen, J. G. Snijders and T. Ziegler, *J. Comp. Chem.*, 2001, **22**, 931-967.
6 C. Fonseca Guerra, J. G. Snijders, G. te Velde and E. J. Baerends, *Theor. Chem. Acc.*, 1998, **99**, 391-403.
7 M. Gaus, Q. Cui and M. Elstner, *J Chem Theory Comput*, 2012, **7**, 931-948.
8 Release 2017a, The MathWorks, Inc., Natick, Massachusetts, United States.
9 E. Van Lenthe and E. J. Baerends, *J Comput Chem*, 2003, **24**, 1142-1156.
10 A. D. Becke, *Phys Rev A Gen Phys*, 1988, **38**, 3098-3100.
11 J. P. Perdew, *Phys Rev B Condens Matter*, 1986, **33**, 8822-8824.
12 V. P. Nicu, J. Neugebauer, S. K. Wolff and E. J. Baerends, *Theor. Chem. Acc.*, 2007, **119**, 245-263.
13 J. Shen, C. Zhu, S. Reiling and R. Vaz, *Spectrochim. Acta A*, 2010, **76**, 418-422.
14 M. A. J. Koenis, Y. Xia, S. R. Domingos, L. Visscher, W. J. Buma and V. P. Nicu, *Chem. Sci.*, 2019, **10**, 7680-7689.
15 M. A. J. Koenis, O. Visser, L. Visscher, W. J. Buma and V. P. Nicu, *J Chem Inf Model*, 2020, **60**, 259-267.
